# Detecting the relevance to performance of whole-body movements

**DOI:** 10.1038/s41598-017-15888-3

**Published:** 2017-11-15

**Authors:** Daisuke Furuki, Ken Takiyama

**Affiliations:** Department of Electrical and Electronic Engineering, Tokyo University of Agriculture and Technology, Koganei-shi, Tokyo 184-8588 Japan

## Abstract

Goal-directed whole-body movements are fundamental in our daily life, sports, music, art, and other activities. Goal-directed movements have been intensively investigated by focusing on simplified movements (e.g., arm-reaching movements or eye movements); however, the nature of goal-directed whole-body movements has not been sufficiently investigated because of the high-dimensional nonlinear dynamics and redundancy inherent in whole-body motion. One open question is how to overcome high-dimensional nonlinear dynamics and redundancy to achieve the desired performance. It is possible to approach the question by quantifying how the motions of each body part at each time point contribute to movement performance. Nevertheless, it is difficult to identify an explicit relation between each motion element (the motion of each body part at each time point) and performance as a result of the high-dimensional nonlinear dynamics and redundancy inherent in whole-body motion. The current study proposes a data-driven approach to quantify the relevance of each motion element to the performance. The current findings indicate that linear regression may be used to quantify this relevance without considering the high-dimensional nonlinear dynamics of whole-body motion.

## Introduction

Sophisticated pitchers can throw a ball toward a desired location. We can grasp a cup in a cupboard while standing in our daily life. These goal-directed movements are fundamental to not only sports, art, and music but also daily living. The nature of goal-directed movements is of current interest in the fields of behavioral science^1,2^, engineering^3,4^, sports science^5^, and neuroscience^6,7^. Our comprehension of how these movements occur is deepened by focusing on two-dimensional arm-reaching movements when seated and when the wrist posture is fixed^7–9^. The investigation of these types of movements enables the exclusion of redundancy (i.e., associating one joint configuration with one endpoint), precise definition of movement error, and investigation of how our neural system controls nonlinear body dynamics^8^.

In contrast, goal-directed whole-body movements are required in daily life and particularly in most sports, such as ball throwing in baseball, basketball, or football; ball kicking in soccer; ball hitting in tennis; and grasping an opponent’s uniform in judo. The objective of goal-directed whole-body movements is to achieve the desired motion performance by overcoming the high-dimensional nonlinear dynamics and redundancy that are inherent in whole-body movements. High-dimensional nonlinear dynamics are associated with these movements because a substantial number of body parts participate in whole-body motion, which causes the dynamics of whole-body motion to be not only nonlinear but also high-dimensional^10^. Redundancy indicates that the number of body parts is more than the minimum necessary to achieve movement goals; as a result, an infinite number of movement patterns may lead to an identical result^11^, and there is no simple one-to-one relationship between whole-body motion and desired performance. Despite the high-dimensional nonlinear dynamics and redundancy of this motion, the desired performance is achieved by somehow overcoming these complexities.

A fundamental question is how we overcome high-dimensional nonlinear dynamics and redundancy. To improve our understanding of goal-directed movements, it may be helpful to investigate various kinds of goal-directed movements in addition to arm-reaching movements. The current study approaches the question in a data-driven manner. We propose a method to detect how the motion of each body part at each time point contributes to performance in observed whole-body motion data and performance data. For example, in a case that involves throwing a ball toward a target, it is uncertain how many times shoulder motion is as relevant as elbow motion to achieve the desired throwing result. It is also unclear whether motion at the time of ball release is as relevant as a motion that occurs 20 msec before the ball is released. If these points were clarified, it would be possible to quantitatively analyze how we control each body part to overcome high-dimensional dynamics and redundancy, deepen our understanding of the nature of goal-directed movements, and clarify how we should modify our movements to achieve desired whole-body movements, thereby enabling the design of efficient training methods. It would also indicate how we should focus on the motions of opponents to predict their movement results, which may improve performance in interpersonal competition. However, high-dimensional nonlinear dynamics and redundancy cause difficulties in the identification of an explicit relationship between the motion element (the motion of each body part at each time point) and performance. The present study thus proposes a data-driven method to detect the relevance of each motion element to movement performance.

Several previous studies have attempted to detect the relevance of particular motion elements to performance. In ball throwing, the timing of finger rotation relative to the timing of the rotation of other joints was more correlated with movement performance than with other motion^12^. A correlation analysis clarified the relation between performance and motion data of only one motion element; however, whole-body motion simultaneously involves multiple motion elements that are correlated with each other (e.g., elbow motion is not independent of shoulder motion). Particularly in goal-directed whole-body movements, methods other than the correlation coefficient should be used to elucidate the relevance of particular movements while simultaneously considering non-independent motion elements. Another previous study attempted to identify the performance-relevant and performance-irrelevant motion elements in Frisbee throwing^13^. The study assumed the averaged motion data across all trials to be the most performance-relevant motion element; however, this assumption is valid only in a limited number of situations. Because whole-body motion involves redundancy, identical results may be generated by an infinite number of motion elements; therefore, the averaged motion data do not always represent the most performance-relevant motion element. For example, in ball throwing, overarm and sidearm throws may produce identical results; however, the averaged motion data across these throwing motions do not have a specific meaning. It thus remains unclear how to quantify the relevance of motion elements to performance in goal-directed whole-body movements in a manner that overcomes the high-dimensional nonlinear dynamics and redundancy of our body dynamics.

Machine-learning techniques may provide a potential approach to analyze whole-body motion data. Recent studies have utilized machine-learning techniques to segment motion data into data obtained while walking and data obtained while running automatically^14^ or for fall detection^15^. Some machine-learning techniques have been shown to work successfully in segmenting sequences of time-series motion data into different motion patterns by focusing on non-goal-directed movements; however, a limited number of studies utilized machine-learning techniques for goal-directed movements. One previous study relied on regression methods to detect the relevance of motion elements to performance in finger movements^16^. This study focused on only the part of the finger that was evidently relevant to a constrained keystroke (e.g., a regression analysis was applied not to all fingers but only to the index finger when the subject was instructed to make a keystroke using that finger) and did not consider timing information. The regression methods used in any study should be evaluated based on the predictive power to avoid overfitting (complicated results are preferred in fitting)^17^; however, the study did not clarify the predictive power obtained. Thus, it is unclear whether certain regression methods work well to detect the relevance of motion elements to performance. The conditions under which relevance may be detected with high precision and whether the same method may be utilized to detect the relevance of several different movement patterns also remain unknown. Thus, it remains unclear how to detect the relevance of motion elements to performance, particularly for goal-directed whole-body movements.

Here, we propose a data-driven approach to detect the relevance of motion elements that takes advantage of the nonlinearity and high-dimensionality of whole-body motion. Because whole-body motion follows high-dimensional and nonlinear dynamics, it may be expected that the identification of an explicit relation between dynamics and movement performance will be difficult; thus, the current study does not focus on this relation. In contrast, it focuses on the identification of the explicit relation in a data-driven manner, thereby proposing a supervised method in which the inputs are time-series data of the whole-body motion trajectory ***x*** and the outputs are low-dimensional movement performance (e.g., if the number of data acquisition markers is 10 and the length of the time series is 1,000 time frames, then ***x*** is 10,000 data points, and movement performance is two-dimensional because an object is thrown toward a two-dimensional target). Because the nonlinearity of whole-body motion is embedded in the high-dimensional motion trajectory data and there is an evident causal relation between motion trajectory and movement performance, the motion trajectory data ***x*** may include a sufficient amount of information to predict movement performance. When ***x*** is appropriately high-dimensional, and a nonlinear function of movement performance, the use of linear regression $y={\boldsymbol{xw}}$ enables the prediction of performance with high accuracy^17^, where $y$ and ***w*** are predicted movement performance and the weight value that indicates how each motion element is relevant to movement performance, respectively. The current study thus proposes a linear regression method that may be used to detect the relevance of each motion element to movement performance. The linear relationship enables the user to clearly and directly analyze the relevance of each motion element to performance based on the value of ***w***. To detect the relevance, the current study uses linear regression and predictive power.

First, we assess whether ridge regression^18^, a linear regression method, may be used to determine the relevance of motion elements to a performance by focusing on a goal-directed whole-body throwing motion in which movement performance (endpoint of throwing objects) and motion data may be simultaneously recorded. We mainly utilize predictive power to evaluate the linear models to avoid the problem of overfitting. Second, we determine the difference between skilled subjects and unskilled subjects based on the relevance of the motion elements to performance, which may suggest how sophisticated performance can be achieved. Third, we investigate the conditions under which ridge regression works well. Fourth, we compare ridge regression to another type of linear regression method, a nonlinear regression method, and a classification method to determine whether ridge regression may work well to detect the relevance. Fifth, we investigate how movement performance should be defined in the detection of relevance. Sixth, we assess the universality of the ridge regression method for detecting relevance by analyzing a whole-body jumping motion. Finally, we investigate efficient feature values for detecting the relevance of motion elements to performance - whether joint angle or segment position is effective for detection.

## Results

### General framework

The present study mainly used linear regression to determine the relevance of motion elements to movement performance in whole-body movements, as subsequently indicated.

1 $$\begin{array}{rcl}{y}_{k} & = & {w}_{0}+\sum _{i=1}^{N}\,\sum _{t={t}_{0}}^{{t}_{1}}({w}_{i,t}^{{\rm{p}},x}{p}_{i,t,k}^{{\rm{x}}}+{w}_{i,t}^{{\rm{p}},y}{p}_{i,t,k}^{{\rm{y}}}+{w}_{i,t}^{{\rm{p}},z}{p}_{i,t,k}^{{\rm{z}}}+{w}_{i,t}^{{\rm{v}},x}{v}_{i,t,k}^{{\rm{x}}}+{w}_{i,t}^{{\rm{v}},y}{v}_{i,t,k}^{{\rm{y}}}+{w}_{i,t}^{{\rm{v}},z}{v}_{i,t,k}^{{\rm{z}}})\\  & = & {w}_{0}+{{x}}_{{k}}{w},\end{array}$$

where ${y}_{k}$ is the predicted performance at the $k$ th trial, ${w}_{0}$ is a bias term, $N$ is the number of focused body parts, ${t}_{0}$ is the number of initial time frames to be analyzed, ${t}_{1}$ is the number of final time frames to be analyzed, ${p}_{i,t,k}^{{\rm{x}}}$, ${p}_{i,t,k}^{{\rm{y}}}$, and ${p}_{i,t,k}^{{\rm{z}}}$ are position data at the *i* ^th^ body part, the *t* ^th^ timing and $k$ th trial in x-, y-, and z-coordinates, respectively, and ${v}_{i,t}^{{\rm{x}}}$, ${v}_{i,t}^{{\rm{y}}}$, and ${v}_{i,t}^{{\rm{z}}}$ are velocity data. ${w}_{i,t}^{{\rm{p}},x}$ indicates how ${p}_{i,t}^{{\rm{x}}}$ is relevant to movement performance, and the other $w$ indicates how each motion element is relevant to performance. When ${w}_{i,t}^{{\rm{p}},x}=0$, the position on the $x$-axis of the *i* ^th^ body part at the *t* ^th^ time frame is not relevant to movement performance; when $|{w}_{i,t}^{{\rm{p}},x}|\, > 0$, it is relevant to movement performance with the strength of the relevance $|{w}_{i,t}^{{\rm{p}},x}|$. If ${w}_{i,t}^{{\rm{p}},x} > 0$ (${w}_{i,t}^{{\rm{p}},x} < 0$), a largerpositive position data for the *x*–coordinate resulted in a larger positive (negative) ${y}_{k}.{{\boldsymbol{x}}}_{k}=$ $({p}_{\mathrm{1,}{t}_{0},k}^{{\rm{x}}},\,\mathrm{...,}\,{p}_{\mathrm{1,}{t}_{1},k}^{{\rm{x}}},\,\mathrm{...,}\,{p}_{N,{t}_{1},k}^{{\rm{x}}},\,\mathrm{...,}\,{p}_{N,{t}_{1},k}^{{\rm{y}}},\,\mathrm{...,}$ ${p}_{N,{t}_{1},k}^{{\rm{z}}},\,\mathrm{...,}\,{v}_{N,{t}_{1},k}^{{\rm{x}}},\,\mathrm{...,}\,{v}_{N,{t}_{1},k}^{{\rm{y}}},\,\mathrm{...,}\,{v}_{N,{t}_{1},k}^{{\rm{z}}})\in {{\bf{R}}}^{1\times (N\times ({t}_{1}-{t}_{0}+\mathrm{1))}}$ and ${\boldsymbol{w}}=({w}_{\mathrm{1,}{t}_{0}}^{{\rm{p}},x},\,\mathrm{...,}\,{w}_{\mathrm{1,}{t}_{1}}^{{\rm{p}},x},\,\mathrm{...,}\,{w}_{N,{t}_{1}}^{{\rm{p}},x},\,\mathrm{...,}\,{w}_{N,{t}_{1}}^{{\rm{p}},y},\,\mathrm{...,}$ ${{w}_{N,{t}_{1}}^{{\rm{p}},z},\mathrm{...,}{w}_{N,{t}_{1}}^{{\rm{v}},x},\mathrm{...,}{w}_{N,{t}_{1}}^{{\rm{v}},y},\mathrm{...,}{w}_{N,{t}_{1}}^{{\rm{v}},z})}^{T}\in {{\bf{R}}}^{(N\times ({t}_{1}-{t}_{0}+\mathrm{1))}\times 1}$, where ${(\cdot )}^{T}$ indicates the vector transpose. The relevance $w$ is a hidden and unknown variable; thus, it should be estimated based on the obtained data.

To determine each $w$, the present study mainly used ridge regression, in which ${w}_{0}$ and each ${w}_{i,t}$ were estimated to minimize the cost function

2 $$E=\frac{1}{2}\sum _{k=1}^{K}{({d}_{k}-{y}_{k})}^{2}+\frac{\lambda }{2}{{\boldsymbol{w}}}^{T}{\boldsymbol{w}},$$

where ${d}_{k}$ represents the data on the performance at the *k* ^th^ trial, $\lambda$ is a regularization parameter, and $K$ is the number of trials to be analyzed. Ridge regression enables the user to estimate $w$ tolerant to observation noise in an analytically tractable form (i.e., one with low computational complexity) by overcoming the problem of collinearity. The first term on the right-hand side in equation (2) represents the prediction error between ${d}_{k}$ and ${y}_{k}$ across all trials; the second term represents the squared norm of the weight value, and the regularization parameter $\lambda$ determines the balance between the minimization of prediction error and the norm. $\lambda$ was determined to minimize the prediction error based on 10-fold cross-validation. If it was impossible to predict the movement performance ${d}_{k}$, $\lambda$ was set to a sufficiently large value such that almost all ${w}_{i,t}$ were estimated to be 0 and ${w}_{0}$ was estimated to be the averaged value of ${y}_{k}$; ${w}_{i,t}=0$ and ${w}_{0}=\frac{1}{K}{\sum }_{k=1}^{K}{d}_{k}$. In contrast, if it was possible to predict the movement performance, $\lambda$ was set to an appropriate value such that each ${w}_{i,t}$ and ${w}_{0}$ were estimated to minimize the cost function. The present study measured whether ridge regression worked well based on the difference between the predictive power with estimated ${w}_{i,t}$, ${w}_{0}$ and the predictive power with ${w}_{i,t}=0$, ${w}_{0}=\frac{1}{K}{\sum }_{k=1}^{K}{d}_{k}$.

The current study defined movement performance data ${d}_{k}$ as the data indicating how the endpoint of throwing objects (in experiments 1 and 3) deviated from the target point (e.g., center of target board) or how the jumping height (in experiment 2) deviated from the target height (e.g., 30 cm-height vertical jump); therefore, ${y}_{k}$ indicates predicted deviation, and ***w*** indicates how relevant each motion element is to the deviation from the target point or height. Although the current study focused on the deviation from the target point or height (i.e., desired motion), linear regression is not restricted to those conditions.

### The predictive power of the linear regression method for a whole-body throwing motion

**Figure 1:**
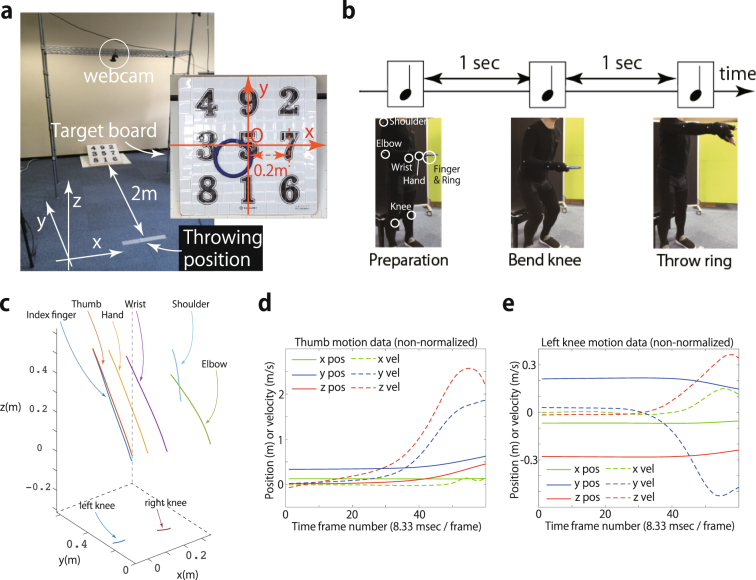
Experimental setting and representative motion data in the ring-throwing experiment. (**a**) Experimental setup. A target board was placed two meters from the throwing position. The throwing position was indicated by a white tape. A webcam was set to record the ring position; the x-, y-, and z-coordinates of the motion data were defined as corresponding to the transverse, sagittal, and coronal planes, respectively. Inset: an example of the ring position recorded by the webcam. The x-axis, y-axis, and origin O of the movement performance were defined as indicated in the inset. (**b**) Subjects threw the ring toward the center of a target board according to beep sounds for which the inter-sound-interval was 1 sec. The first beep represented a preparation signal to indicate the initiation of one trial. The subjects were instructed to bend their knees after they heard the second beep and to throw the ring at the time of the third beep. The use of this method decreased the trial-to-trial variability in the movement time. Motion capture markers were attached to the participants’ thumb, index finger, hand, wrist, elbow, shoulder, left knee, and right knee. The markers are indicated in the figure by white circles. (**c**) Non-normalized movement trajectory data of a typical subject in the x-, y-, and z-coordinates. The figure indicates the position data averaged across 100 trials. (**d**,**e**) Temporal profiles of the non-normalized position and velocity of the markers attached to the thumb and the left knee. The profiles were obtained by averaging the position data across 100 trials.

The subjects were instructed to throw a ring (133 g weight) toward the center of a 2-meter (m)-distant target (i.e., the location of the numeral 5 on the target board [Fig. 1a]). All the participants were naïve to the ring throwing. To control the movement time, the present study utilized beep sounds; the first beep signaled the initiation of the trial, the second beep indicated the signal to bend the knees, and the subjects were instructed to throw the ring at the third beep (Fig. 1b). The inter-beep interval was 1 second. The subjects (nine male subjects aged 18–31 years (mean 21.1 and standard deviation 4.5); eight subjects for three sessions and one subject for ten sessions) threw the ring more than 100 times per session. Trials in which the ring bounced or slipped on the board were excluded from the analysis. In total, data from 100 trials were analyzed per session.

The movement performance at the $k$th trial, ${d}_{k}$, was defined as the endpoint of the ring. The performance was two-dimensional; ${d}_{{\rm{x}},k}$ and ${d}_{{\rm{y}},k}$, the endpoints on the x-axis and y-axis, respectively, were defined as indicated in Fig. 1a at the $k$ th trial. Ridge regression was independently used for each session to determine ***w*** to predict ${d}_{{\rm{x}},k}$ and ***w*** to predict ${d}_{{\rm{y}},k}$ (i.e., the optimized $\lambda$ for predicting ${d}_{{\rm{x}},k}$ was different from that for predicting ${d}_{{\rm{y}},k}$ in each session).

The present study also measured motion data for the right shoulder, the right elbow, the right wrist, the back of the right hand, the right thumb, the right index finger, and the right and left knees using a motion capture system (Optitrack Flex 13, NaturalPoint Inc., Corvallis, Oregon, Fig. 1b). Data on the ring position were used only for the detection of the release timing. The motion data of 60 time frames, in which the 60th time frame corresponded to the release timing, were used in the analysis. The 60 time frames were discretized into 12 time bins, each of which consisted of 5 time frames, to determine how the motion at each time point was related to performance. In the ring throwing, the number of markers was 8, the coordinates of the motion data were 3 in number (x-, y-, and z-coordinates), and the position and velocity of each marker and coordinate were analyzed. As a result, the total number of data points in the motion data in each time bin was $5\times 8\times 3\times 2=240$. The motion data in each data point were normalized to satisfy a mean of 0, and standard deviation of 1 (e.g., $\frac{1}{K}{\sum }_{k=1}^{K}\,{p}_{i,t,k}^{{\rm{x}}}=0$ and $\frac{1}{K}{\sum }_{k=1}^{K}\,{({p}_{i,t,k}^{{\rm{x}}})}^{2}=1$) except for Figs 1, 4a, 6, 7d, 8b, and 8d. Thus, the current study considered a regression in which the input data (motion data) were 240 data points and the target data (performance data) were 1-dimensional.

**Figure 2:**
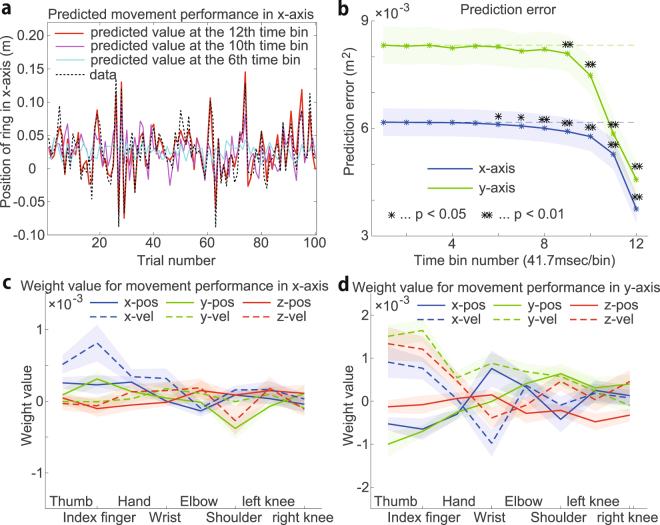
Detection of the relevance of each motion element to performance. (**a**) Representative predicted movement performance on the x-axis using ridge regression at the 12th time bin (red line), the 10th time bin (magenta line), and the 6th time bin (cyan line). The black dotted line indicates the performance data. (**b**) Relation between the prediction error and time bin. The horizontal and vertical axes indicate the time bin number and prediction error (mean squared error), respectively. Each time bin consists of five time frames or 41.7 msec. The 60th time frame in the 12th time bin corresponds to the time of release. The blue and green asterisks indicate the prediction error averaged across 34 sessions on the x-axis and y-axis, respectively. The shaded areas indicate the standard error. The prediction error in each session was evaluated by 10-fold cross-validation using the optimized regularization parameter $\lambda$. The black asterisks indicate significant differences at the p $<$ 0.01 (double black asterisks) or p $<$ 0.05 (single black asterisk) levels in the Wilcoxon signed-rank test between the prediction error at the time bin and that with ${w}_{i,t}=0$ and ${w}_{0}=\frac{1}{K}{\sum }_{k=1}^{K}{d}_{k}$. If there was no efficient relationship between the movement performance $t$ and the motion data ${\bf{x}}$, the weight values in ridge regression were estimated as ${w}_{i,t}=0$ and ${w}_{0}=\frac{1}{K}{\sum }_{k=1}^{K}{d}_{k}$. Thus, we compared the prediction error at each time bin and that with the weight values. (**c**,**d**) Estimated weight values at the 12th time bin. The weight values in each session were averaged as ${\bar{w}}_{i}=\frac{1}{5}{\sum }_{t=56}^{60}{w}_{i,t}$ because each time bin includes 5 time frames. The solid and dotted lines indicate the averaged ${\bar{w}}_{i}$ across all sessions with respect to the position and velocity, respectively. The x-, y-, and z-coordinates are shown in blue, green, and red, respectively. The shaded areas indicate the standard error of ${\bar{w}}_{i}$.

Ridge regression could predict movement performance on both the x- and y-axes (Fig. 2a indicates the predicted performance on the x-axis). Notably, Fig. 2a indicates the predicted performance. The data from the 100 trials were divided into the data from 90 trials and the data from 10 trials. The 90-trial data were used to estimate each $w$, and the 10-trial data were used for the prediction (i.e., the red, magenta, and cyan lines indicate the predicted values without reference to the performance data). Prediction performance is a sophisticated measure that may be used to avoid overfitting and makes it possible to obtain validated ***w*** values^17,19^. The dynamics of whole-body motion are highly complicated; however, the predictive power indicated that ridge regression, a linear regression model, could be used to predict performance in the whole-body ring throwing movements.

### Detecting the relevance of motion elements to performance

Ridge regression indicated that the ring-throwing performance could be predicted four time bins (approximately 160 ms) before ring release (Fig. 2b). Figure 2b indicates the relationship between the prediction error (means $$\pm $$ s.e.m. for 34 sessions) and the time bin number. The single and double asterisks indicate significant differences with p $$ < 0.05$$ and p $$ < 0.01$$, respectively, in the Wilcoxon signed-rank test between the prediction error at the time bin and the prediction error calculated with each $${w}_{i,t}$$ equal to 0 and $${w}_{0}=\frac{1}{K}{\sum }_{k=1}^{K}{d}_{k}$$ (these $$w$$ corresponded to the case in which there was no relation between motion and performance data, e.g., at the 1st time bin).

The use of linear regression methods enables a discussion of the relevance of motion elements to performance based on ${w}_{i,t}$, that is, how the motion of the *i*th body part (the body part to which the *i*th data acquisition marker was attached) at the *t*th time frame is relevant to predict the movement performance. Figures 2c and d indicate the estimated ${w}_{i,t}$ based on the motion data at the 12th time bin. The horizontal and vertical axes indicate the marker numbers (or body part) and ${\bar{w}}_{i}=\frac{1}{5}{\sum }_{t=56}^{60}{w}_{i,t}$ (the averaged ${w}_{i,t}$ across all the time frames at the 12th time bin), respectively. The blue, green, and red lines indicate ${\bar{w}}_{i}^{{\rm{x}}}$, ${\bar{w}}_{i}^{{\rm{y}}}$, and ${\bar{w}}_{i}^{{\rm{z}}}$ for the position (solid line) or velocity (dotted line), respectively (means $\pm$ s.e.m. for 34 sessions). In Fig. 2c, the blue dotted lines at the thumb and index finger indicate the largest and second largest values, which suggest that both the thumb and index finger are relevant to the movement performance on the x-axis. Furthermore, ${\bar{w}}_{i}$ for both the thumb and index finger exhibit positive values, which indicates that the rightward faster thumb and index finger velocities in the x-coordinate result in more rightward-deviated movement performance on the x-axis. Ridge regression thus enables a discussion of the relevance of motion elements to performance.

**Figure 3:**
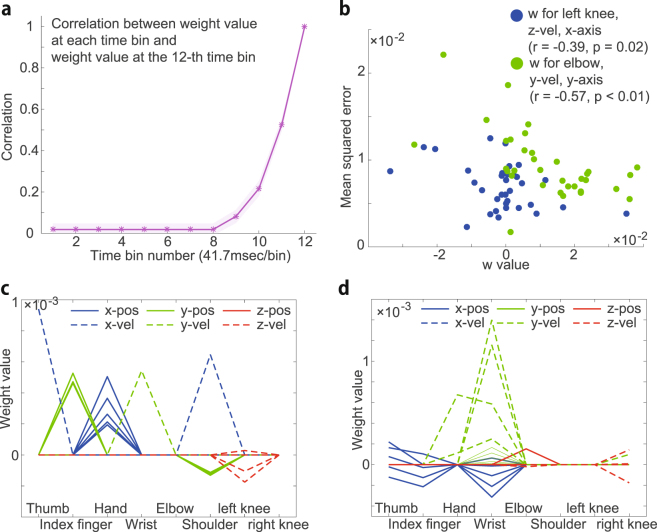
Properties of the weight values. (**a**) Relation between the estimated weight values and movement timing. The horizontal axis indicates the time bin number, and the vertical axis indicates the correlation between the weight values estimated at the 12th time bin and the values estimated at each individual time bin. The solid line and shaded area indicate the mean and standard error, respectively. (**b**) The relationship between a typical weight value and performance (mean squared error). The blue and green dots indicate the relation between the weight value for left knee motion at the 12th time bin and the performance on the x-axis in each session and the relation between the weight value for elbow motion at the 12th time bin and the performance on the y-axis in each session, respectively. (**c**,**d**) The estimated weight values ${w}_{i,t}$ at the 12th time bin that had significant correlations with performance (p $<$ 0.05).

Predictive power was maximal at the 12th time bin, which contained the time frames when the release occurred, and was lower at the 11th, 10th, and earlier time bins. There are at least two possibilities for the lower predictive power at the earlier time bins; ${w}_{i,t}$ estimated at the 12th time bin was downregulated at the 11th, 10th, and earlier time bins, or the structure of ${w}_{i,t}$ estimated at the 12th time bin was different from that of ${w}_{i,t}$ estimated at 11th, 10th, and earlier time bins. In the former case, the correlation coefficient between ${w}_{i,t}$ estimated at the 12th time bin and ${w}_{i,t}$ estimated at each time bin should be high across all time bins. In contrast, in the latter case, the correlation should have different values at each time bin. Figure 3a, which indicates the correlation between ***w*** estimated at the 12th time bin and ***w*** estimated at each time bin, partially supports the latter possibility. Because body motion is temporally smooth, ***w*** estimated at the 12th time bin should be correlated with ***w*** estimated at the 11th time bin. Taken together, partially but not completely overlapped motion elements were estimated to be relevant to movement performance in each time bin.

### Factors of skilled and unskilled performance

Even sophisticated athletes showed not only trial-to-trial variability in performance but also day-to-day variability in performance (e.g., an athlete demonstrates the best performance on one day, but he/she demonstrates the worst performance on the following day). Because the estimated ${w}_{i,t}$ in Figs. 2c and d indicated how relevant each motion element was to trial-to-trial variability in performance, it remained unclear whether the estimated ${w}_{i,t}$ indicated which motion element was relevant to day-to-day variability in performance. In other words, because the differences between skilled and unskilled performance remained unclear, we assessed the relationship between ${w}_{i,t}$ and session-to-session variability in performance.

Figures 3c and d show the estimated ${w}_{i,t}$ at the 12th time bin that had a significant correlation (p $<$ 0.05) to the mean squared error in the ring-throwing motion on the x-axis and y-axis, respectively (mean for 34 sessions). Because subjects were instructed to throw the ring toward the center of the target board $({d}_{{\rm{x}}},{d}_{{\rm{y}}})=\mathrm{(0,0)}$, the mean squared error $\frac{1}{K}{\sum }_{k=1}^{K}{d}_{k}^{2}$ could be regarded as a measure of performance in each session. ${w}_{i,t}$, which did not have any significant correlation to the mean squared error, was plotted as 0. There were up to 5 lines in each ${w}_{i}$ because each time bin included five time frames. Although the index finger was associated with the largest value of ${\bar{w}}_{i}$ in Figs. 2c and d, ${w}_{i,t}$ related to the motion of the index finger was not significantly correlated with session-to-session variability in performance. Some unremarkable ${w}_{i,t}$ in Figs. 2c and d were significantly correlated with the variability (e.g., ${w}_{i,t}$ related to the motion of the shoulder was significantly correlated with the session-to-session variability in performance on the x-axis, and ${w}_{i,t}$ related to the motion of the wrist was significantly correlated with the session-to-session variability in performance on the y-axis). Figure 3b indicates the representative relation between ${w}_{i,t}$ and performance in each session. The position of the left knee in the z-coordinate was correlated with the mean squared error on the x-axis (r = −0.39, p = 0.02), and the velocity of the wrist in the y-coordinate was correlated with the mean squared error on the y-axis (r = −0.57, p $<$ 0.01). These results indicated that linear regression revealed not only the trial-to-trial variability in performance but also the day-to-day variability in performance; thus, the estimated ${w}_{i,t}$ may suggest the factors of skilled and unskilled performance.

### Reliability of linear regression - individual differences

**Figure 4:**
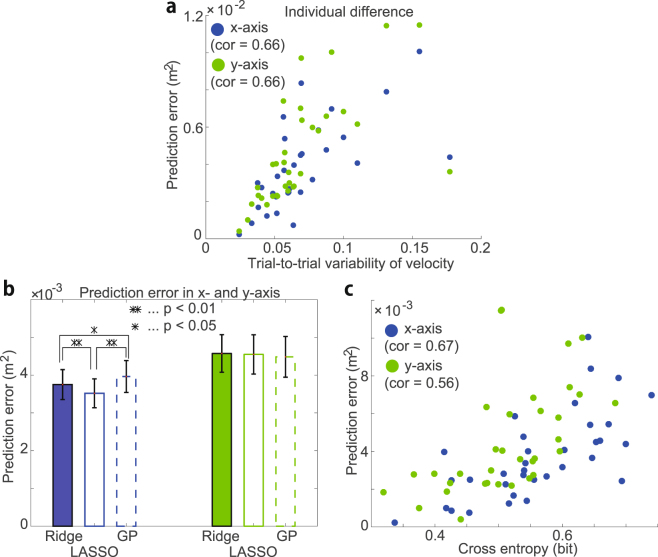
Validation of the ridge regression. (**a**) Relation between the trial-to-trial variability in the non-normalized velocity and prediction error at the 12th time bin (a detailed description of the variability is provided in the Online Methods section). The blue and green dots indicate the variability and prediction error in each session for the x-axis and y-axis, respectively. The variability and prediction error were correlated with each other (r = 0.66 and p $<$ 0.01 the on the x- and y-axes). (**b**) Prediction error of ridge regression, lasso, and Gaussian process regression on the x-axis (blue bars) and y-axis (green bars). The filled bars, blank bars, and blank bars with dotted lines indicate the means $\pm$ s.e.m. by ridge regression, lasso, and Gaussian process regression, respectively. Black asterisks indicate significant differences at the level of p $<$ 0.01 (double black asterisks) or p $<$ 0.05 (single black asterisk) in the Wilcoxon signed-rank test. (**c**) Relation between the prediction error of ridge regression and cross-entropy calculated by logistic regression at the 12th time bin in each session. Large cross-entropy indicates frequent failure of classification. The blue and green dots indicate the cross-entropy and prediction error in each session for the x-axis and y-axis, respectively. Those values were correlated with each other (r = 0.67 and p $<$ 0.01 on the x-axis and r = 0.56 and p $<$ 0.01 on the y-axis).

The detection of the relevance of motion elements to performance was reliable only when the predictive power was reliable; thus, the conditions under which linear regression works well should be clarified. The present study investigated the correlation between the prediction error and the trial-to-trial variability in the non-normalized motion data at the 12th time bin (a detailed description of the variability is provided in the Materials and Methods section). For performance on the x-axis, there were significant correlations between the variability in the position and the prediction error (r = 0.383, p = 0.0255) and between the variability in the velocity and the prediction error (r = 0.660, p $<$ 0.01, Fig. 4a). For performance on the y-axis, there was no significant correlation between the variability in the position and the prediction error (p = 0.0791); however, there was a significant correlation between the variability in the velocity and the prediction error (r = 0.657, p $<$ 0.01, Fig. 4a). Thus, linear regression may be reliable for subjects whose velocity is stable across trials.

### Reliability of linear regression - comparison with other methods

We determined whether linear regression is valid for detecting the relevance of the motion element to performance by comparing the predictive power of ridge regression to the predictive power of other methods, including Lasso^20^ (least absolute shrinkage and selection operator), Gaussian process regression^21^, and logistic regression^22^. Lasso is another linear regression method, Gaussian process regression is a nonlinear regression method, and logistic regression is a linear classification method.

Other methods may be better than ridge regression for detecting the relevance of motion elements to performance; however, the present study indicated that there was no systematic improvement in prediction performance using other methods compared with ridge regression (Fig. 4b, means $\pm$ s.e.m. for 34 sessions). The lasso method yielded a sparse solution in which most ${w}_{i,t}$ were estimated to be 0. The lasso method exhibited better predictive power than ridge regression and Gaussian process regression in predicting performance on the x-axis (p $<$ 0.01); however, the present study indicated that there was no improvement in predicting performance on the y-axis (p = 0.844 for ridge regression and p = 0.9251 for Gaussian process regression). Gaussian process regression did not exhibit any improvement over ridge regression (p = 0.017 for ridge regression on the x-axis), but the mean prediction error was larger for Gaussian process regression than for ridge regression (p = 0.447 on the y-axis). Because there were no systematic differences in the results achieved using ridge regression, another linear regression method (lasso), and a nonlinear regression method (Gaussian process regression), ridge regression may represent an efficient approach to detect the relevance of motion elements to performance in the current study.

Logistic regression is a classification method. For example, it classifies whether the ring position on the x-axis is positive or negative, and it predicts whether the binary movement performance ${s}_{x}$ will be 0 or 1(${s}_{x}=1$ when ${d}_{{\rm{x}}} > 0$, and ${s}_{x}=0$ otherwise). Figure 4c indicates the predictive power of logistic regression and ridge regression. The predictive power of the classification method was evaluated based on the cross-entropy: ${\sum }_{k=1}^{K}{s}_{k}\,\mathrm{log}\,{f}_{k}-{\sum }_{k=1}^{K}\mathrm{(1}-{s}_{k})\mathrm{log}(1-{f}_{k})$, where ${s}_{k}$ represents the binary data and ${f}_{k}$ is the estimated probability for ${s}_{k}=1$ at the *k* ^th^ th trial. When ${f}_{k}$ was appropriately estimated to satisfy ${f}_{k}=1$ for ${d}_{k}=1$ and ${f}_{k}=0$ for ${d}_{k}=0$, the cross-entropy equaled 0. In contrast, when ${f}_{k}$ was not appropriately estimated to satisfy ${f}_{k}=0.5$ for ${d}_{k}=1$ and ${f}_{k}=0.5$ for ${d}_{k}=0$, the cross-entropy equaled $K\,\mathrm{log}\,2$. Thus, the smaller cross-entropy resulted in better performance in classification.

The regression and classification methods exhibited similar predictive powers (the correlations between the prediction error of ridge regression and the cross-entropy of logistic regression across all sessions at the 12th time bin were r = 0.672 and p $<$ 0.01 for the x-axis and r = 0.555 and p $<$ 0.01 for the y-axis). The magnitude of each ${w}_{i,t}$ was different for ridge and logistic regression as a result of the difference in the definition of movement performance (a continuous value for regression and a binary value for classification); however, there was a correlation between the ${\bar{w}}_{i}$ estimated by ridge regression and that estimated by logistic regression at the 12th time bin ($r=0.839$ and p $<$ 0.01 for the x-axis and $r=0.910$ and p $<$ 0.01 for the y-axis). This finding indicates that similar relevance of motion elements to performance may be detected by both regression and classification methods; thus, ridge regression was shown to be an efficient approach to detect the relevance of motion elements to performance in the current study.

### Dependence on a coordinate of movement performance

The current study focused on detecting the relevance of motion elements to performance on the x- and y-axes defined in Fig. 1a; however, another definition of movement performance is the distance between the target and the ring endpoint position, ${d}_{{\rm{r}}}=\sqrt{{d}_{{\rm{x}}}^{2}+{d}_{{\rm{y}}}^{2}}$. Using the classification method, it was possible to define the movement performance as success when ${d}_{{\rm{r}}} < 0.1$ and failure otherwise, for example. Thus, we investigated the predictive power of regression and classification methods when movement performance was defined in r-coordinates.

**Figure 5:**
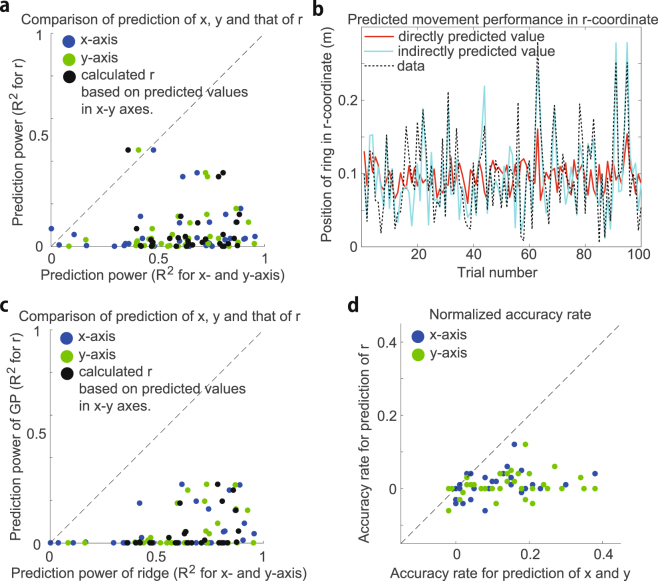
Comparison of the predicted movement performance on the x- and y-axes and r-coordinates. (**a**) ${{\rm{R}}}^{2}$ for the predicted movement performance on the x- (blue dots) and y-axes (green dots) and r-coordinates (data in r-coordinates were calculated as ${t}_{{\rm{r}},k}=\sqrt{{d}_{{\rm{x}},k}^{2}+{d}_{{\rm{y}},k}^{2}}$). Each dot indicates ${{\rm{R}}}^{2}$ in each session. The black dots indicate ${{\rm{R}}}^{2}$ for the predicted movement performance in r-coordinates and that in r-coordinates by calculating ${y}_{{\rm{r}},k}=\sqrt{{y}_{{\rm{x}},k}^{2}+{y}_{{\rm{y}},k}^{2}}$ using the predicted performance on the x- and y-axes. (**b**) Predicted performance in r-coordinate by directly applying ridge regression in the r-coordinate (red line) and indirectly calculating the predicted performance on the x- and y-axes (cyan line). The black dotted line indicates actual data in the r-coordinate. (**c**) Predictive power of movement performance in the r-coordinate by Gaussian process regression, a nonlinear method. The horizontal axis indicates ${{\rm{R}}}^{2}$ for the predicted movement performance on the x-axis (blue dots) and the y-axis (green dots) using ridge regression, and the vertical axis indicates that in the r-coordinate using the Gaussian process. The black dots indicate the same values as in (**a**). (**d**) Normalized accuracy rate for the prediction of movement performance on the x-axis (blue dots), y-axis (green dots), and r-coordinates by logistic regression, a classification method. The normalized accuracy rate was calculated by subtracting the accuracy rate at the 1st time bin from that at the 12th time bin.

Figure 5 indicates the predictive power of movement performance on the x- and y-axes and r-coordinates at the 12th time bin using ridge regression, Gaussian process regression, and logistic regression. The predictive power measured by the coefficient of determination ${{\rm{R}}}^{2}$ for both the x- and y-axes was significantly higher than that for the r-coordinate (p $<$ 0.01 for both the x-axis (blue dots in Fig. 5a) and y-axis (green dots in Fig. 5a), Wilcoxon signed-rank test), which indicates that movement performance should be discussed in terms of the x- and y-axes. The indirectly predicted performance on the r-coordinate based on the predicted performance on the x- and y-axes (i.e., ${y}_{{\rm{r}}}=\sqrt{{y}_{{\rm{x}}}^{2}+{y}_{{\rm{y}}}^{2}}$) was better than the directly predicted performance on the r-coordinate (black dots in Fig. 5a, p $<$ 0.01, Wilcoxon signed-rank test). Figure 5b also indicates worse predictive performance in the r-coordinates (red line) and better predictive performance when the performance in the r-coordinates was indirectly predicted via the predicted performance on the x- and y-axes (cyan line).

Because there was a nonlinear transformation from ${d}_{{\rm{x}}}$ and ${d}_{{\rm{y}}}$ to ${d}_{{\rm{r}}}$, one potential approach to predict ${d}_{{\rm{r}}}$ is nonlinear transformation of the motion data. However, the predictive power of the performance on the x- and y-axes by ridge regression was better than that in the r-coordinate by Gaussian process regression (Fig. 5c, p $<$ 0.01 on the x-axis (blue dots in Fig. 5c) and y-axis (green dots in Fig. 5c) and indirectly predicted performance in the r-coordinate (black dots in Fig. 5c), Wilcoxon signed-rank test). Classification by logistic regression also did not work well in classifying ${d}_{{\rm{r}}} < 0.1$ (Fig. 5d; the predictive power was better on the x- and y-axes (blue and green dots) than that in the r-coordinates, p $<$ 0.01, Wilcoxon signed-rank test). These findings indicate that movement performance must be appropriately defined to detect the relevance of each motion element to the performance.

### The generality of linear regression in another type of motion

**Figure 6:**
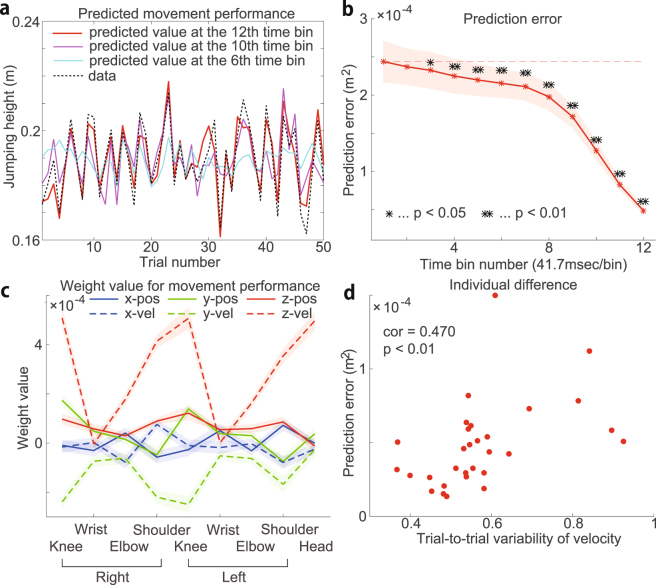
Detection of the relevance of each motion element to performance in the jumping experiment. (**a**) Predicted jumping height using ridge regression at the 12th time bin (red line), the 10th time bin (magenta line), and the 6th time bin (cyan line). The black dotted line indicates the jumping height data. (**b**) Relation between the prediction error and time bin. The red asterisks indicate the averaged prediction error across 30 sessions. The shaded areas indicate the standard error. The prediction error in each session was evaluated by 10-fold cross-validation using the optimized regularization parameter $\lambda$. The black asterisks indicate significant differences at the level of p $<$ 0.01 (double black asterisks) or p $<$ 0.05 (single black asterisk) in the Wilcoxon signed-rank test between the prediction error at the time bin and that with ${w}_{i,t}=0$ and ${w}_{0}=\frac{1}{K}{\sum }_{k=1}^{K}{d}_{k}$. (**c**) The estimated weight values ${\bar{w}}_{i}$ at the 12th time bin. The solid and dotted lines indicate the averaged ${\bar{w}}_{i}$ across all sessions with respect to position and velocity, respectively. The x-, y-, and z-coordinates are shown in blue, green, and red, respectively. The shaded areas indicate the standard error of ${\bar{w}}_{i}$. (**d**) Relation between the trial-to-trial variability in the non-normalized velocity data and prediction error at the 12th time bin. The variability and prediction error were correlated with each other (r = 0.47 and p $<$ 0.01 on the x- and y-axes).

The linear regression method works well for ring-throwing motions; however, it should be clarified whether the method also works well for other types of motion. The current study investigated the generality of the applicability of linear regression to motion analysis by investigating another motion, namely a jumping motion (seven subjects, with 50 jumps per session; five subjects for two sessions and two subjects for ten sessions; all the participants were naïve to the jumping motion.). The detailed experimental settings are described in the Materials and Methods section. Linear regression was shown to work well for a jumping motion (Fig. 6), similar to the ring-throwing motion. Thus, these findings indicate that the linear regression method may be widely utilized to detect the relevance of motion elements to performance for several types of goal-directed whole-body movements.

### Comparison between joint angle and segment position

**Figure 7:**
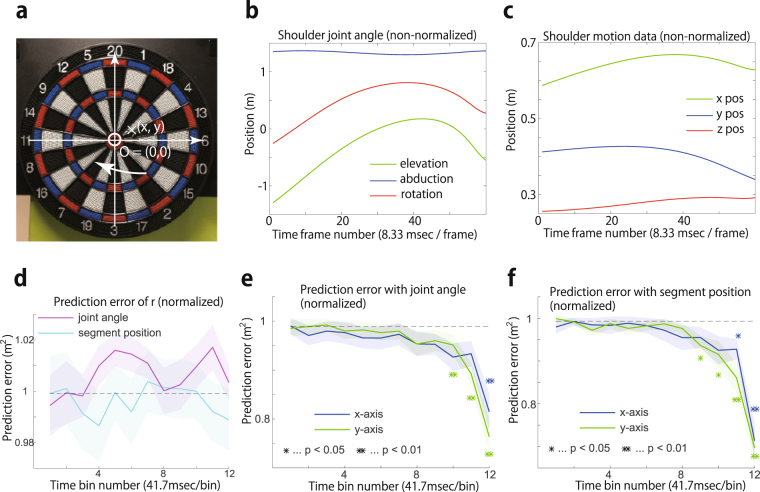
Dart-throwing experiment. (**a**) Definition of performance. The x-axis, y-axis, and origin O of the movement performance were defined as indicated. (**b**) Non-normalized shoulder joint angle data of a typical subject. The figure shows the joint angle data averaged across 50 trials. (**c**) Non-normalized movement trajectory data of a typical subject in the x-, y-, and z-coordinates. The figure shows the shoulder position data averaged across 50 trials. (**d**) Relation between the time bin and the prediction error in the r-coordinate by directly applying ridge regression in the r-coordinate. (**e**) Relation between the prediction error and time bin based on the joint angle. Blue and green lines indicate the average prediction error across 20 sessions. The shaded areas indicate the standard error. The prediction error in each session was evaluated by 10-fold cross-validation using the optimized regularization parameter $\lambda$. The double and single asterisks indicate significant differences at the level of p $<$ 0.01 or p $<$ 0.05 in the Wilcoxon signed-rank test between the prediction error at the time bin and that with ${w}_{i,t}=0$ and ${w}_{0}=\frac{1}{K}{\sum }_{k=1}^{K}{d}_{k}$. Blue and green asterisks indicate the differences in prediction error for the performance on the x- and y-axes, respectively. (**f**) Relation between prediction error and time bin based on segment position.

The selection of feature values affects predictive performance in linear regression. Although the current study mainly focused on segment position (e.g., the position of the shoulder, elbow, or other body parts), most previous studies relied on joint angle^12,13,16^. We thus compared predictive performance using either segment position or joint angle in the dart-throwing experiment (see the Methods section for details). Ridge regression enabled us to predict performance using either joint angle (Fig. 7e) or segment position (Fig. 7f). The number of joint angles in each joint in the current study were those of the shoulder (Fig. 7b), one for the elbow, three for the wrist, and two for the index finger. The segment position in the current study was that of the shoulder, elbow, wrist, hand, and index finger in the x-, y-, and z-coordinates. In the dart-throwing experiment and the case of ridge regression, the current study found a slightly better predictive power using segment position than using joint angle at the 12th time bin (p $<$ 0.05 with Wilcoxon signed-rank test).

We further investigated whether joint angle enabled us to predict movement performance in the r-coordinate (Fig. 7d) because nonlinear computation to calculate joint angles could be expected to improve the predictive performance. There was no significant difference between the predictive performance using joint angle and segment position (p = 0.5257 with Wilcoxon signed-rank test). Further, there was no significant difference between predictive performance using the motion data at the 1st time bin and at the 12th time bin (p = 0.7369 with Wilcoxon signed-rank test in using joint angle data and p = 0.4553 with Wilcoxon signed-rank test using segment position data). Movement performance should thus be defined on the $x-$ and $y-$ axes when applying linear regression to detect the relevance of motion elements to performance.

## Discussion

We propose a linear regression method that may be used to detect the relevance of motion elements to performance in goal-directed whole-body movements. High-dimensionality, nonlinearity, and redundancy cause difficulty in detecting relevance; however, we showed that a linear regression method could be used to predict movement performance (Figs. 2a and b) and detect the relevance of motion elements (Figs. 2c and d), despite the finding that the method did not consider nonlinearity. The use of linear regression provides advantages in analyzing the relevance of motion elements based on the weight value ${w}_{i,t}$ regarding how the motion of the *i* ^th^ body part at the *t* ^th^ time point is related to movement performance (Figs. 2c and d). It should be noted that the linear regression is applicable to various kinds of motions when both motion data ***x*** and performance data ***d*** are available. In addition, we described the difference between skilled and unskilled performance (Figs. 3b–d), clarified the conditions under which linear regression could be used to detect the relevance of motion elements to performance (Fig. 4a), validated the use of ridge regression to detect the relevance by comparing it to other regression and classification methods (Figs. 4b and c), showed that the relevance should be detected on the x- and y-axes rather than the r-coordinates (Fig. 5), demonstrated the generality of the linear regression method by applying the method to another goal-directed whole-body movement (Fig. 6), and showed slightly better predictive performance using segment position than using joint angle (Fig. 7).

Our methods detected how relevant each motion element was to performance by considering motion data of multiple segments that were correlated to each other. A previous study discussed the performance-relevant motion elements based on correlation coefficients^12^. The correlation coefficient considers only a single motion element or multiple motion elements that are independent of each other. Our linear regression can be considered an extended method to simultaneously consider multiple correlated motion elements. Some previous studies that utilized an uncontrolled-manifold (UCM) framework estimated performance-relevant components as averaged motion data across trials^13^. Our linear regression estimated the relevance of each motion element to performance based on data without assuming that the averaged motion data were the most relevant to the performance. In contrast to the UCM framework, our linear regression did not explicitly consider the relationship between each joint, although the correlation of each motion element could be considered to estimate ***w***. A possible extension is to utilize our linear regression technique to estimate a performance-relevant motion element(s) and apply UCM analysis for the estimated element(s).

Another possible method for detecting the relevance of motion elements to performance is to simulate whole-body motion based on a Newtonian equation of the whole body. For the experimental simulations (e.g., ring-throwing experiment), we need to explore the appropriate temporal varying torque of each joint (e.g., knee, shoulder, elbow, finger, or other body parts); finding an appropriate torque sequence and simulating how a slight difference in each torque results in a difference in ring throwing is time-consuming. If it is possible to utilize an inverse-dynamics technique, we imagine that the simulations would be easier. However, we need to measure the force applied to a ring in experiment 1 and the force applied to a dart in experiment 3, which requires developing new devices for each experiment. Our data-driven technique enables us to discuss the relevance of each motion element to performance with measured motion and performance data.

Related previous works have analyzed of the relevance of motion elements to performance based on correlations or relative relationships among multiple joints^12,16^. In motion data, cross-correlations between body parts should be calculated to consider how each body part is moved in a coordinated manner. However, the number of the cross-correlation to be estimated in whole-body motion may be substantial because the numbers of body parts and time frames are large (e.g., when the number of markers is 8 and the number of time frames is 60, the dimension of the cross-correlation is $64\times 40=2560$ even when the maximum temporal lag is 40). If it is possible to conduct an experiment with a large number of trials, there is no problem with including the correlation in the regression or classification methods; nevertheless, because performing many repeated whole-body movements may cause fatigue or attention loss, it is practically difficult to obtain a substantial amount of data at one time and include the correlation in the regression method. The current study thus focused on each body part and the timing of each movement independently.

All the participants in our experiments were naïve to the ring-throwing, jumping, and dart-throwing motions. Although they practiced each motion, there is a possibility that they improved their skill in each trial. If their skill improved in each trial, the relevance of each motion element to performance would change in each trial, and it would be impossible to predict performance based on cross-validation. Because linear regression could predict performance based on cross-validation, the improvement in skill was not evident at least in each session.

There were no significant differences in predictive performance among the ridge regression, lasso, and Gaussian process regression (Fig. 4b). However, the number of trials may have affected the performance of these methods. Lasso or other sparse machine-learning techniques frequently exhibit better performance than other methods when the number of trials is sufficiently smaller than the dimension of the input data. In contrast, Gaussian processes or other nonlinear techniques frequently exhibit better predictive performance than other methods when the number of trials is sufficiently large; however, the interpretation may be more complicated than when linear methods are used. An appropriate method for detecting the relevance of motion elements to performance likely needs to be selected based on the individual learning task; however, ridge regression worked well in the current study.

Whole-body motions have frequently been analyzed based on methods used to classify whether the subjects are running or walking^14^ and to detect falls^15^. The classification methods worked well in the current study; logistic regression classified whether ${d}_{{\rm{x}}} > 0$ or ${d}_{{\rm{x}}} < 0$ and whether ${d}_{{\rm{y}}} > 0$ or ${d}_{{\rm{y}}} < 0$ (Fig. 4c). However, it was impossible to determine whether the motion in each trial was successful (e.g., ${d}_{{\rm{r}}}=\sqrt{{d}_{{\rm{x}}}^{2}+{d}_{{\rm{y}}}^{2}} < 0.1$ or not) using logistic regression. Because regression worked and predicted more precise information (e.g., $y$ = 0.1, −0.05, …), the regression method was better than the classification method for detecting the relevance of motion elements to performance.

The dynamics of whole-body movements are highly nonlinear; thus, it remains unclear how linear regression may be used to measure the relevance of motion elements to performance. One potential explanation is related to the nonlinearity and high-dimensionality of motion data. A linear regression model, referred to as a motor primitive framework in this context, has been successfully used in modeling motor learning with a goal-directed arm-reaching movements^23–27^. In this framework, a complicated motor command $u$ required to achieve the required movements is generated from the linear weighted sum of complex neural activities $A$; $u=WA$ and $W$ are modified to minimize the movement error between the actual hand position and the desired movement position. When $A$ is a nonlinear function of the desired movement position and appropriately high-dimensional, nonlinear motor commands can be generated by appropriate linear combinations of nonlinear neural activities, which has been theoretically validated in the framework of a basis function network^28,29^. Correspondingly, in the current study, motion data $x$ could be a nonlinear function of movement performance (e.g., the endpoint of the ring toss) because the dynamics of whole-body motion are nonlinear. Furthermore, the motion data were appropriately high-dimensional (240 data points in motion data for 1-dimensional movement performance in the current study). Thus, an appropriate linear summation ***xw*** could predict the actual movement performance, which resulted in an appropriately estimated ***w*** that represented the relevance of motion elements to performance.

It remains unclear whether the detected relevance of motion elements to performance might be used as efficient feedback signals to enhance movement performance in sports and rehabilitation. This issue provides a basis for fascinating future studies.

## Materials and Methods

### Ethics statement

All participants were clearly informed of the experimental procedures in accordance with the Declaration of Helsinki, and all participants provided written informed consent before initiation of the experiment. All procedures were approved by the ethics committee of Tokyo University of Agriculture and Technology.

### Ring-throwing experiment

Nine healthy volunteers (all males aged 18–31 years [mean 21.1 and standard deviation 4.5]) participated in this study. The participants were instructed to stand and make throwing movements with their right arms toward the center of a target board (Fig. 1a). All the participants were naïve to the ring throwing. The target board was 2 m away from the participants. The participants were also instructed to perform the throwing movements according to three beep sounds (refer to the Main Text). The use of the beep sounds reduced the trial-to-trial variability in the movement time. The participants grasped the ring by putting their thumbs and index fingers on the tape attached to the ring, which made the distance between the marker on the ring and that on the thumb holding the ring constant. The position of the ring was also measured, and the moment of release was defined as the time at which the distance between the ring and the thumb exceeded 0.001 m on the basis of the distance at the beginning of the throwing motion.

### Jumping experiment

Seven healthy volunteers (all males aged 20–32 years [mean 22.7 and standard deviation 4.6]) participated in this study. All the participants were naïve to the jumping motion. Markers were attached to the participants’ right and left feet, right and left knees, navel (center), right and left wrists, right and left elbows, right and left shoulders, and head. The participants were instructed to stand next to tape attached to the floor and perform 0.3-m-high jumping movements according to three beep sounds, similar to the ring-throwing experiments. Tape was attached 0.3 m above the head, and the participants were instructed to jump so that their heads touched the tape. When the participants failed to touch the tape, the experimenters instructed them to jump higher. The moment of release was defined as the first time frame in which the averaged position of the markers on the right and left feet on the z-axis exceeded 0.001 m on the basis of the distance at the beginning of the jumping motion. The jumping height was determined as the maximal height of the averaged position of the markers on the right and left feet on the z-axis.

### Dart-throwing experiment

Five healthy volunteers (all males aged 22–32 years [mean 26.0 and standard deviation 4.2]) participated in this study. All the participants were naïve to the dart-throwing motion. Markers were attached to the participants’ shoulder (three), elbow (two), wrist (two), hand (one), and index finger (one) to calculate joint angles. The participants were instructed to stand 2.44 m away from a 1.73-m-high dart board following the official rules and to throw a dart according to three beep sounds. In the dart-throwing experiment, participants were instructed to throw a dart at the time of the third beep. The moment of release was defined as the first time frame in which the distance between the dart and index finger exceeded 0.001 m on the basis of the distance at the beginning of the throwing motion. We used a 10th order lowpass FIR filter with a cutoff frequency of 12 Hz for the position data of each marker. The endpoint of the dart was normalized to satisfy $\frac{1}{K}{\sum }_{k=1}^{K}{d}_{k}=0$ and $\frac{1}{K}{\sum }_{k=1}^{K}{d}_{k}^{2}=1$.

### Regression and classification

For regression and classification, the current study utilized MATLAB(R2016a). The MATLAB package *glmnet* ^30^ was used for ridge regression, lasso, and logistic regression, and the package *gpregress* was used for Gaussian process regression. In logistic regression, the current study utilized the ${L}_{2}$ regularization $\frac{\lambda }{2}{\sum }_{i,t}{w}_{i,t}^{2}$ or ridge-logistic regression.

### Definition of trial-to-trial variability in velocity

The trial-to-trial variabilities in the position and velocity in Figs 4a and 7d were calculated as the averaged value across all time frames and marker numbers at the 12th time bin (i.e., $\frac{1}{15N}{\sum }_{i=1}^{N}{\sum }_{t=56}^{60}({{\rm{Var}}}_{k}({v}_{i,t}^{{\rm{x}}})+{{\rm{Var}}}_{k}({v}_{i,t}^{{\rm{y}}})+{{\rm{Var}}}_{k}({v}_{i,t}^{{\rm{z}}}))$, where ${{\rm{Var}}}_{k}$ indicates the variance calculated across all trials).
